# Left Ventricular Assist Device as a Bridge to Candidacy in End-stage Chagas Cardiomyopathy

**DOI:** 10.5935/abc.20180095

**Published:** 2018-07

**Authors:** Fernando Antibas Atik, Claudio Ribeiro Cunha, Renato Bueno Chaves, Marcelo Botelho Ulhoa, Vitor Salvatore Barzilai

**Affiliations:** Instituto de Cardiologia do Distrito Federal, Brasília, DF – Brazil

**Keywords:** Chagas Cardiomyopathy, Heart Failure, Shock, Cardiogenic, Hypertension, Pulmonary, Extracorporeal Circulation

## Introduction

Chronic Chagas cardiomyopathy manifests late after *Trypanosoma cruzi* infection, and it is still one of the major causes of end-stage heart failure in Latin America.^1^ Published experiences^2,3^ with heart transplantation for chronic Chagas disease have shown the feasibility and efficacy of this therapy, being the disease reactivation not a major concern when appropriately diagnosed and timely treated.^4^

Nevertheless, the need for some sort of mechanical circulatory support on the waiting list has progressively increased, according to the International Society for Heart and Lung Transplantation Registry annual report.^5^ Since the experience with mechanical circulatory support in Latin America is very limited, and biventricular systolic failure is common in Chagas cardiomyopathy, many unanswered questions regarding modalities of support need to be elucidated.

This case report describes the successful implant of an axial flow left ventricular assist device in a patient with end-stage heart failure with severe biventricular failure secondary to Chagas cardiomyopathy.

## Case Report

A 26-year-old male, with a history of long-standing heart failure had multiple hospital admissions in the past year despite optimal medical management. The diagnosis of end-stage heart failure due to Chagas cardiomyopathy was confirmed by serology a while ago, and an implantable cardioverter defibrillator was used for sudden death secondary prevention. Echocardiography revealed a severely dilated left ventricle (end-diastolic diameter of 72 millimeters), with severely depressed function (ejection fraction of 18%) and 4+ mitral regurgitation. The right ventricle also exhibit severe dysfunction with 3+ tricuspid regurgitation, tricuspid annular plane systolic excursion of 15, and right ventricular systolic pressure of 65 mmHg. The patient has been followed up in a different city of ours by another cardiology team. At this point, he has never been considered for heart transplantation. 

Nonetheless, the patient was admitted in the emergency room with cardiogenic shock, in Interagency Registry for Mechanically Assisted Circulatory Support (INTERMACS) level 2. He was initially managed with the use of two inotropes, intra-aortic balloon pump and hemodialysis. No temporary or durable mechanical assist devices were available at this hospital.

A right heart catheterization revealed low cardiac output (cardiac index of 0.9 L/min/m^2^, with systolic pulmonary pressure of 70 mmHg, transpulmonary gradient of 16 mmHg and pulmonary vascular resistance of 6 Wood units. Filling pressures were elevated (central venous pressure and pulmonary wedge pressure of 30 mmHg).

**Figure 1:**
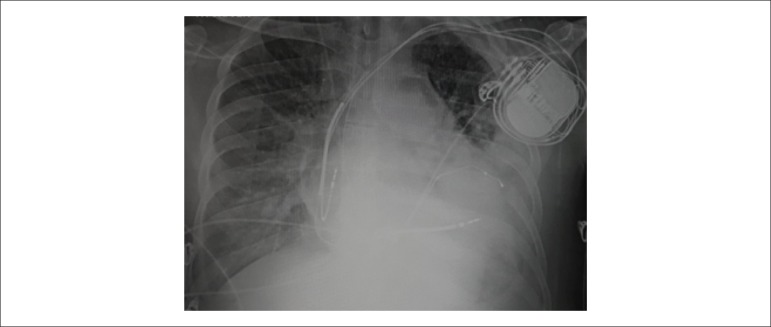
Chest radiography after ECLS implantation.

The patient was transferred to our hospital for heart transplantation assessment. At admission, he had sudden hemodynamic instability that deteriorated into cardiac arrest. Cardiopulmonary resuscitation measures were effective, but circulation was maintained with escalating doses of vasopressors. A percutaneous venous arterial extracorporeal life support (ECLS) (Maquet Getinge^TM^, Germany) through the femoral vessels was inserted as a bridge to decision strategy. Hemodynamics stabilized, vasopressors were discontinued, tissue perfusion indices normalized, and the patient neurologic status was intact. He was extubated on the next day, renal function normalized, an aggressive diuresis allowed a twelve-liter negative fluid balance in the following five days (Figure 1).

Eighteen days after ECLS initiation, the patient was submitted to an axial flow left ventricular assist device (HeartMate II, Abbott Laboratories^TM^, Chicago, IL) implantation with ECMO explant under median sternotomy with cardiopulmonary bypass.

**Figure 2:**
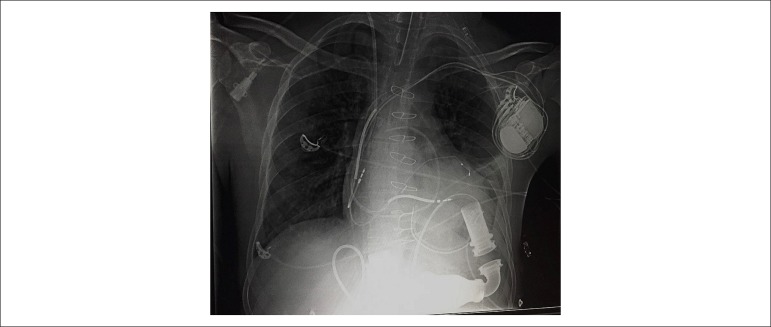
Chest radiography after left ventricular assist device implantation.

Postoperatively (Figure 2), the patient had mediastinal bleeding requiring surgical revision; coagulopathy and pericarditis. A transient right ventricular dysfunction required a five-day administration of intravenous inotropic support, aggressive diuresis and oral pulmonary vasodilators. He was eventually discharged home on postoperative day 35 in fair condition, requiring rehabilitation due to malnutrition and muscular weakness.

Sixteen months later, he is in functional class I with unremarkable recovery except for a single episode of hemolysis that was treated with intravenous heparin. Pump has functioned well with no evidence of failure or thrombosis. Late right heart failure was not an issue, and his exercise performance is excellent. Echocardiography revealed mild tricuspid regurgitation and right ventricular systolic pressure of 30 mmHg. At this point, the patient does not manifest interest in being transplanted.

## Discussion

The present report illustrates that, in patients with Chagas cardiomyopathy with severe biventricular failure, a left ventricular assist device (and not necessarily a biventricular support) may be considered as a modality of mechanical circulatory support as a bridge to candidacy or transplantation. Frequent pathologic findings that need to be observed are apical aneurysms, mural thrombi, very thin ventricular walls and complex ventricular arrhythmias refractory to ablation. Destination therapy, in theory, is a possible alternative for those patients that do not present with late right heart failure, which is a possible natural manifestation of the disease.

Since published data is very limited and the experience with mechanical circulatory support in Latin America is scarce, there are no consensus regarding the best strategy. Moreira et al were the first to report the use of paracorporeal devices in Chagas cardiomyopathy, with inconsistent results.^6^ More recently, Kransdorf et al^7^ reported the United States experience on 11 heart transplants for Chagas cardiomyopathy. Three out of 11 patients (27%) had mechanical circulatory support in place at the time of transplant (two patients had paracorporeal devices in biventricular configuration and one patient had a HeartMate II device). Ruzza et al^8^ described the successful support with a total artificial heart prior to heart transplantation. They argue that this approach is justifiable because it allows treatment of extracardiac Chagas disease, and it potentially reduces the infectious burden of the causative organism that may make progress the disease on a heart supported with a device.

This particular case presented cardiogenic shock, fluid overloaded, with a recent cardiac arrest that required the use of venous arterial ECLS. It was very difficult to determine whether the pulmonary hypertension was severe enough to contraindicate the heart transplantation. Therefore, a bridge to candidacy strategy seemed reasonable in this regard. After six months of support, it proved to be effective in reducing the pulmonary vascular resistance making the patient eligible for heart transplantation.
